# Development and evolution of human glutaminyl cyclase inhibitors (QCIs): an alternative promising approach for disease-modifying treatment of Alzheimer's disease

**DOI:** 10.3389/fnagi.2023.1209863

**Published:** 2023-08-03

**Authors:** Daoyuan Chen, Qingxiu Chen, Xiaofei Qin, Peipei Tong, Liping Peng, Tao Zhang, Chunli Xia

**Affiliations:** ^1^School of Bioengineering, Zunyi Medical University, Zhuhai, China; ^2^Fujian Key Laboratory of Translational Research in Cancer and Neurodegenerative Diseases, School of Basic Medical Sciences, Institute of Basic Medicine, Fujian Medical University, Fuzhou, China

**Keywords:** Alzheimer's disease, human glutaminyl cyclase, amyloid-β, pyroglutamate modification, QC inhibitor, PQ912, PBD150

## Abstract

Human glutaminyl cyclase (hQC) is drawing considerable attention and emerging as a potential druggable target for Alzheimer's disease (AD) due to its close involvement in the pathology of AD via the post-translational pyroglutamate modification of amyloid-β. A recent phase 2a study has shown promising early evidence of efficacy for AD with a competitive benzimidazole-based QC inhibitor, PQ912, which also demonstrated favorable safety profiles. This finding has sparked new hope for the treatment of AD. In this review, we briefly summarize the discovery and evolution of hQC inhibitors, with a particular interest in classic Zinc binding group (ZBG)-containing chemicals reported in recent years. Additionally, we highlight several high-potency inhibitors and discuss new trends and challenges in the development of QC inhibitors as an alternative and promising disease-modifying therapy for AD.

## 1. Introduction

Alzheimer's disease (AD) is a complex neurodegenerative disease that is clinically characterized by progressive and irreversible dysfunction of language, memory, and cognition (Association, 2023). AD is the leading cause of dementia in the elderly, representing 60–80% of dementia cases globally (Association, 2023). Projections indicate that the number of people living with dementia around the world will sharply increase from 55 million to 139 million by 2050 (Association, 2023). However, for more than a century, only five drugs have been approved for the symptomatic treatment of AD, and these drugs are incapable of retarding or reversing disease progression. China and the United States have recently approved the mannan oligosaccharide GV-971 (Syed, 2020) and the anti-Aβ antibody aducanumab (Aduhelm) (Dhillon, 2021) as novel disease-modifying treatments for AD, respectively. Nevertheless, both treatments have been questioned for their limited clinical efficacy in clinical trials. Therefore, it is still urgently needed to develop new disease-modifying therapies for the early intervention of AD.

The deposition of senile plaques, dominantly consisting of β-amyloid proteins (Aβs), is one of the pathological hallmarks of AD brains. Full-length Aβ_1 − 40/42_ is generated through the amyloidogenic processing of the amyloid precursor protein (APP) mediated by the β-site APP cleaving enzyme (BACE) and γ-secretase complex (Chen et al., 2017). Compelling evidence showed that the highly hydrophobic and aggregation-prone Aβ_1 − 40/42_ plays an upstream role in the pathological progression of AD via inducing tau hyperphosphorylation, synaptic dysfunction, and neuroinflammation (Selkoe and Hardy, 2016; Lee et al., 2017). Hundreds of Aβ targeting or Aβ-related therapeutic strategies have thus been proposed in the past three decades (van Bokhoven et al., 2021), while unfortunately most of the interventions failed in clinical trials due to limited effects on cognition recovery or unfavorable safety profiles in AD patients.

**Figure 1:**
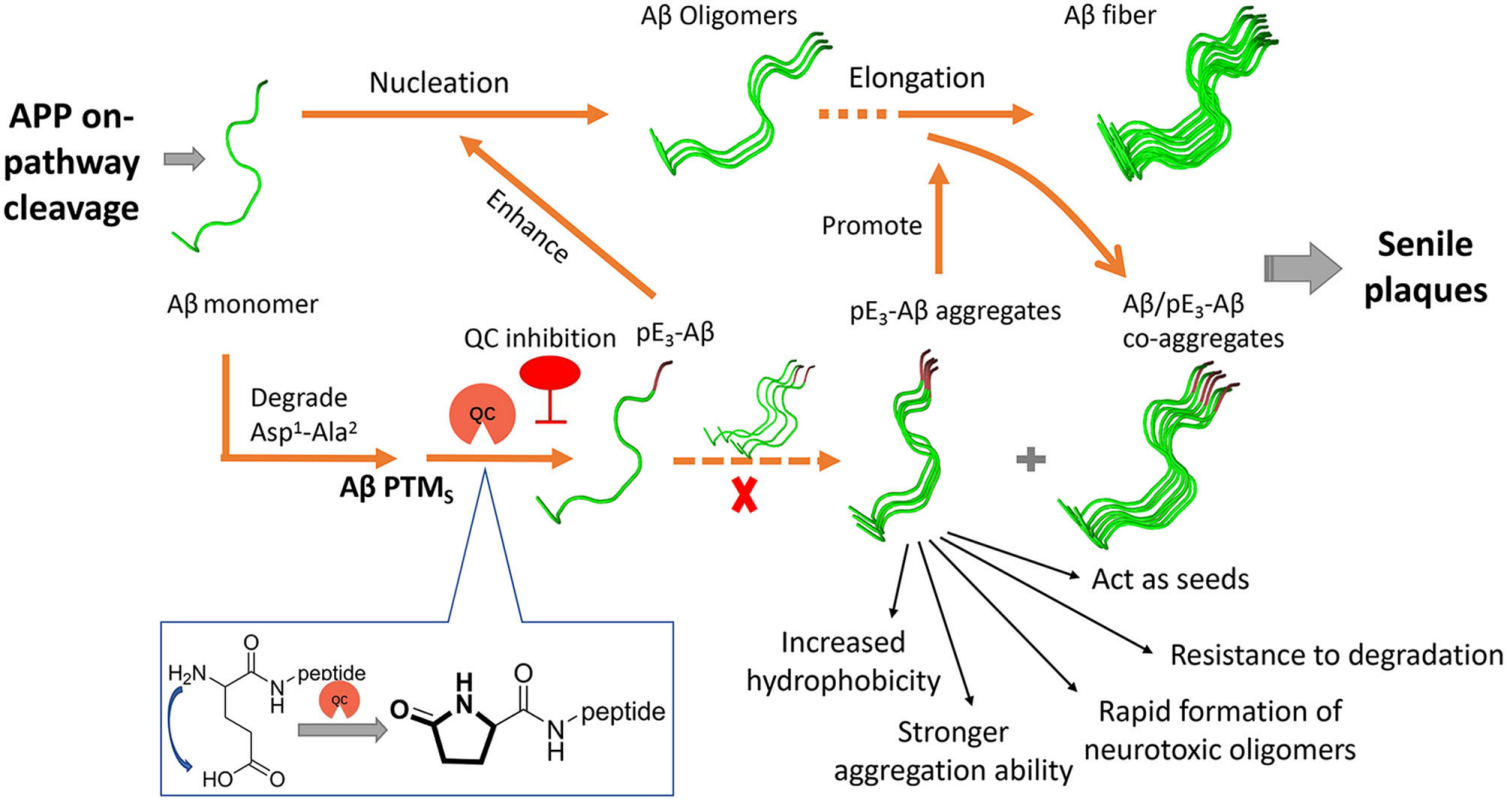
Generation and roles of pE_3_-Aβ in AD and schematic drawing of the QC inhibition approach.

Aβs in the senile plaques are highly diverse and heterogeneous due to various post-translational modifications (PTMs) such as truncations, oxidation, and pyroglutamation (Roher et al., 2017). The continuous failures have prompted researchers to reevaluate the role of PTMs of Aβ in the pathogenesis of AD (Grochowska et al., 2017; Roher et al., 2017). Among the PTMs, the pyroglutamation product pE_3_-Aβ has recently been shown to be closely involved in AD (Figure 1) and is gradually presumed to be a highly desirable biomarker and intervention target (Jawhar et al., 2011a; Bayer, 2022). pE_3_-Aβ is formed through the dehydration and cyclization of the Glu3 residue of the truncated Aβ_3 − 40/42_ under the catalytic action of human glutaminyl cyclase (hQC) (Figure 1) (Schilling et al., 2004; Cynis et al., 2008). It is noteworthy that the release of truncated Aβ_3 − 40/42_ is independent of BACE and may primarily relate to Meprins, members of the “astacin family” of metalloproteinases, which are able to cleave APP after the Ala2 at the N-terminus of the Aβ sequence (Stephan Schilling and Demuth, 2010).

pE_3_-Aβ constitutes a prominent fraction of the total Aβ species in AD brains (Harigaya et al., 2000; Wu et al., 2014) and the critical initiating role of pE_3_-Aβ in AD was supported by several lines of evidence (Figure 1) (Gunn et al., 2010; Nussbaum et al., 2012). First, pE_3_-Aβ has hundreds-fold higher aggregation ability (Schilling et al., 2006) and is much easier to maintain the neurotoxic oligomeric states compared with full-length Aβ (Lee et al., 2014; Gunn et al., 2016; Wulff et al., 2016). It can also act as seeds to accelerate Aβ assembly (Dammers et al., 2017b) and subsequently form denser, more stable, and cytotoxic Aβ/pE-Aβ copolymers than those of Aβ_1 − 40/42_ aggregates (Schilling et al., 2006; Nussbaum et al., 2012). Second, pE_3_-Aβ is perhaps more resistant to aminopeptidase due to the lactam ring in the N-terminus of pE_3_-Aβ (Gontsarova et al., 2008), which contributes to prolonged neurotoxicity *in vivo*. In addition, observations revealed that pE_3_-Aβ acts upstream of the neurotoxic Aβ cascade (Dammers et al., 2017a; Bayer, 2022). It progressively accumulates in the brain at the early stage of AD, even before full-length Aβ aggregation, and subsequently triggers neurodegeneration and ultimately exacerbates the severity of AD pathology and cognition. In a most recent study, donanemab, a pE_3_-Aβ-specific antibody developed by Eli Lilly, significantly cleared amyloid plaques and slowed down cognitive deterioration in patients with mild AD in a phase II trial (Mintun et al., 2021) and met all the primary and secondary endpoints in a phase III trial (TRAILBLAZER-ALZ 4), reducing brain amyloid plaque levels by 65.2% at 6 months compared to baseline (data were shared on 30 November 2022 at the Clinical Trials on Alzheimer's Disease conference). All this evidence strongly supports pE_3_-Aβ as an effective therapeutic target (Perez-Garmendia and Gevorkian, 2013).

QC is intimately associated with the pathology and severity of AD by paralleling the generation of pE-Aβ in the brain (Figure 1) (Morawski et al., 2014), and the deposition of pE-Aβ was found to be restricted to APP/QC co-expression areas (Hartlage-Rübsamen et al., 2018). Furthermore, the expression and enzymatic activity of QC are significantly elevated and are positively correlated with both the accumulation of pE-Aβ and cognition decline in the brains of AD subjects compared with those of age-matched controls (Valenti et al., 2013; Gunn et al., 2021). Besides, both QC knockout and treatment with QC inhibitors (QCIs) significantly rescue the behavioral phenotype and alleviate disease-like pathology in the AD mouse model (Schilling et al., 2008; Jawhar et al., 2011b). Hence, small molecule-based QCIs provide an alternative, promising, and cost-effective therapeutic approach apart from immunotherapy for early-stage AD treatment (Coimbra et al., 2019; Coimbra and Salvador, 2021; Xu et al., 2021). Recently, PQ912 (varoglutamstat), a QC competitive inhibitor developed by Probiodrug AG, passed the clinical phase IIa trial (Scheltens et al., 2018) and is regarded as the proof-of-concept validation of QC.

Over the past two decades, a number of QCIs, including both synthetic and natural compounds, have been discovered. Several reviews focusing on the function of QC and the development of QCIs have been published (Coimbra et al., 2019; Vijayan and Zhang, 2019; Coimbra and Salvador, 2021; Xu et al., 2021; Zhang et al., 2022), while new design and screening strategies have been applied in discovering new QCIs with unique structural characteristics in recent years. Hence, we reexamine the discovery and evolution of QC inhibitors, with a particular interest in classic Zinc binding group (ZBG)-containing chemicals. In addition, we highlight several representative high potent inhibitors as well as the challenges of QCIs as potential disease-modifying therapies for AD.

## 2. Brief functions and structural features of hQC

N-terminal pyroglutamation of proteins is ubiquitously found in a variety of organisms, including bacteria, plants, and animals, and two types of QCs with distinctive structures and catalytic sites have been identified and classified in these organisms so far. Type I QCs are mainly found in plants and bacteria, as exemplified by *Papaya* QC and *Myxococcus xanthus* QC, while type II QCs are primarily present in animals (Taudte et al., 2021), such as *Drosophila melanogaster* QC and human QC (hQC), which share substantial sequence identity and structural similarity (Koch et al., 2012b). Type I QC exhibits a five-bladed β-propeller structure composed of β-sheets and antiparallel β-strands, together with a Ca^2+^-binding motif in the active core (Carrillo et al., 2010), which significantly differs from the α/β topology and Zn^2+^-binding motif in the catalytic center of Type II hQCs (Taudte et al., 2021).

hQC, known as human glutaminyl-peptide acyltransferase (QPCT, EC2.3.2.5), belongs to the acyltransferase family and is abundant in the human brain and neuronal tissues. hQC is broadly expressed in various neurons, including urocortin-1 and cholinergic Edinger-Westphal neurons, as well as locus coeruleus and nucleus basalis Meynert neurons (Morawski et al., 2010). Normally, hQC promotes the maturation of neuropeptides or cytokines such as gonadotropin-releasing hormone (GnRH), thyrotropin-releasing hormone (TRH), and chemokine CCL-2 via catalyzing the cyclization of glutamine residue at the N-terminus of proteins (Cynis et al., 2011; Becker et al., 2016; Vijayan and Zhang, 2019). It was later revealed that QC only shows modest specificity for cyclization of their primary glutaminyl substrates (Seifert et al., 2009), it can also catalyze N-terminal glutamate cyclization (Schilling S. H. et al., 2003; Schilling et al., 2004), which thus provides a close link between QC and AD pathophysiology via the formation of pE-Aβ. Nevertheless, the enzymatic conversion has strikingly different condition preferences, with glutaminyl conversion occurring with an optimum pH of 8.0, whereas glutamyl conversion is favored at a pH of 6.0 (Schilling et al., 2004).

There are two isoforms of QC in humans, namely, the secretory QC (sQC, 361aa, encoded by the QPCT gene located at 2p22.2) and golgi-resident QC (gQC or isoQC, 382aa, encoded by the QPCTL gene located at 19p13.32). sQC is a secreted protein that contains a N-terminal secretion signal, while gQC contains a N-terminal anchor responsible for the retention within the Golgi complex. sQC and gQC share a sequence identity of >45%, have similar catalytic domain sizes, and catalyze the same enzymatic reaction (Stephan et al., 2009), making it uneasy to design iso-specific inhibitors. The discrepancy distribution of sQC and gQC results in the conversion of different substrates and even distinct physiological roles, which was suggested to be beneficial to the complementary function regulation of QC in a non-catalytic specificity manner (Coimbra and Salvador, 2021). As pE-Aβ in humans is mainly catalyzed by sQC rather than gQC *in vivo*, we will focus on the sQC inhibitors for the treatment of AD in this review.

The catalytic domain of sQC contains Zn^2+^ and approximately 330 amino acid residues, exhibiting a globular α/β-fold open-sandwich topology that comprises a central six-stranded β-sheet (among which two were antiparallel) surrounded by two and six α-helices on the opposite sides and flanked by two α-helices at one edge of the β-sheet (Huang et al., 2005; Xu et al., 2021). The catalytic domain has a hydrophobic entrance and a relatively narrow binding pocket. The essential Zn^2+^ is located at the bottom of the active pocket, coordinating with three conservative residues (Asp159, Glu202, and His330) and a water molecule to form a tetrahedral structure, which is necessary for catalysis (Huang et al., 2005). The loop domains near the active center of sQC have certain conformational variabilities that might be affected by N-linked glycosylation (Ruiz-Carrillo et al., 2011); meanwhile, the glycosylation has a limited impact on the overall structure and catalytic activity of sQC but may influence its solubility (Schilling et al., 2002; Ruiz-Carrillo et al., 2011). The crystal structure of hQC also revealed a unique hydrogen-bond network in the active site, formed by five highly conserved residues (Ser160, Glu201, Asp248, Asp305, and His319), within which Glu201 and Asp248 participate in binding to the substrate. When natural substrates or inhibitors enter the catalytic center, the carbonyl of glutamine, glutamate, or other metal binding groups can replace water molecules to coordinate with Zn^2+^ (Huang et al., 2008; Coimbra and Salvador, 2021), thereby catalyzing or inhibiting the cyclization of glutamine and glutamate of the substrates. The structural features, especially the mono-Zn^2+^ binding model, offer the most valuable guidance for the design and discovery of QCIs.

## 3. Development and evolution of QCIs

### 3.1. Rational design and experiment-based QCIs

A metal-chelating group has been initially considered an essential functional component for the construction of QCIs since the identification of hQC as a Zn^2+^-dependent metalloenzyme and the discovery that chelators such as imidazole and its analogs have weak QC inhibitory activity (Schilling S. et al., 2003; Schilling S. H. et al., 2003; Demuth et al., 2004a,b). Probiodrug AG (currently Vivoryon Therapeutics N.V.) was a pioneer in developing high-activity QCIs (Demuth et al., 2004a,b; Buchholz et al., 2008), and as early as 2006, the company contributed the foundational literature for the design and discovery of the first high potent imidazole-containing QC inhibitor via mimicking a tripeptide (Gln-Phe-Ala-NH_2_) substrate (Buchholz et al., 2006). The strategy has been proven to be highly efficient in generating a library of QCIs with K_i_ ranging from nanomolar to micromolar. In particular, the strongest inhibitor **1** (Table 1A), known as PBD150 or PQ50, had an excellent K_i_ of 60 nM (Buchholz et al., 2006). It was unexpected that PBD150 was approximately 19-fold more effective toward sQC than gQC, whereas the co-crystallization of PBD150-sQC complex revealed an almost identical binding mode as observed in PBD150-gQC complex, except for the slightly stronger hydrophobic interaction with Ile303 compared with that of Val324 in gQC (Huang et al., 2011). The binding properties of PBD150 to sQC in solution provide additional evidence that the conformation of PBD150 is susceptible to disruption through protein-protein interactions (Koch et al., 2012a). Surprisedly, replacing imidazole in PBD150 with 5-methyl imidazole leads to a stronger inhibitor **2** (Table 1A), with almost a 10-fold increase in the activity compared with PBD150 (Buchholz et al., 2009). However, the inhibitory activity of **3** (Table 1A) decreased to basal level when connecting with the two methoxy groups on the benzene ring (Tran et al., 2013). Subsequent studies regularly employed comparable substrate-mimicking approaches utilizing an alternative Aβ_3 − 5_ (Glu-Phe-Arg) or used PBD150 and **2** as lead compounds, leading to the identification of inhibitors with shared pharmacophores and an increasingly elucidated structure-activity relationship (SAR).

**Table 1:** Representative design and experiment-based QCIs.

**(A) QCIs with classic motifs A, B, and C**	
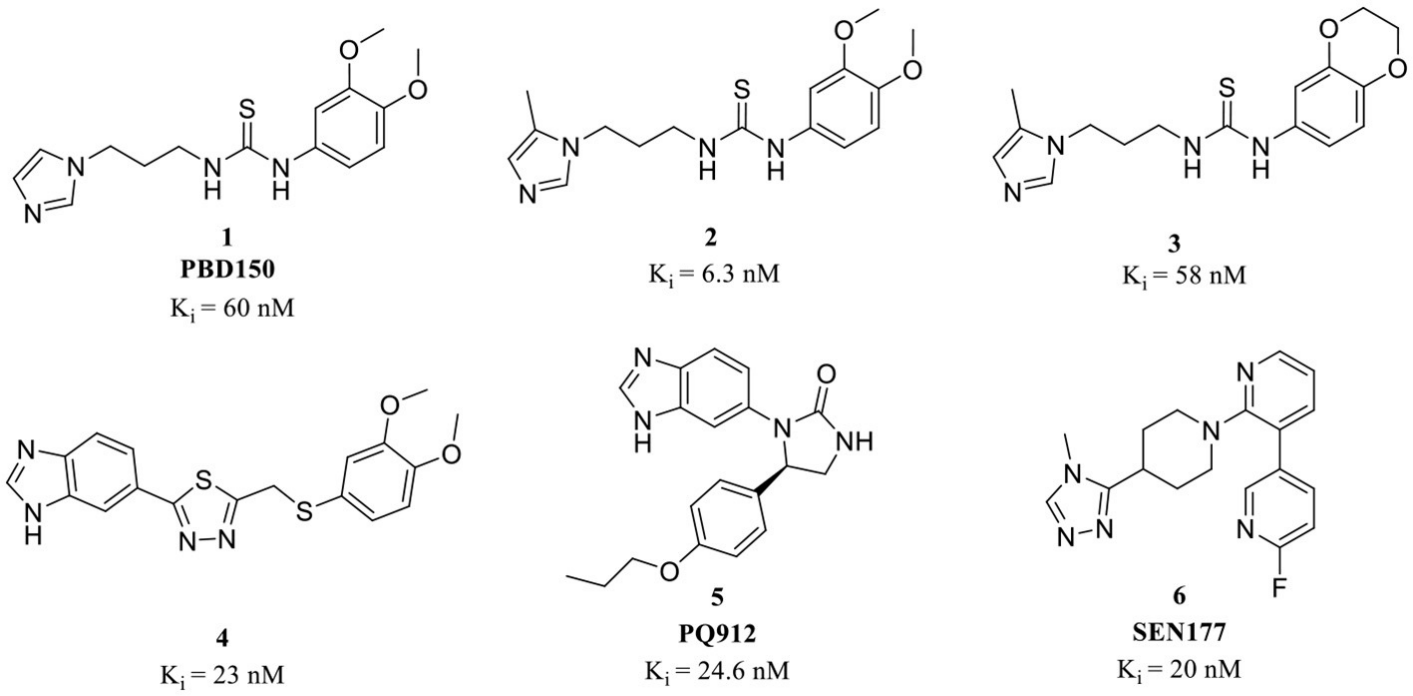	**1** (Buchholz et al., 2006); **2** (Buchholz et al., 2009); **3** (Tran et al., 2013); **4** (Ramsbeck et al., 2013); **5** (Lues et al., 2015); **6** (Pozzi et al., 2018)
**(B) QCIs with extended motif D**	
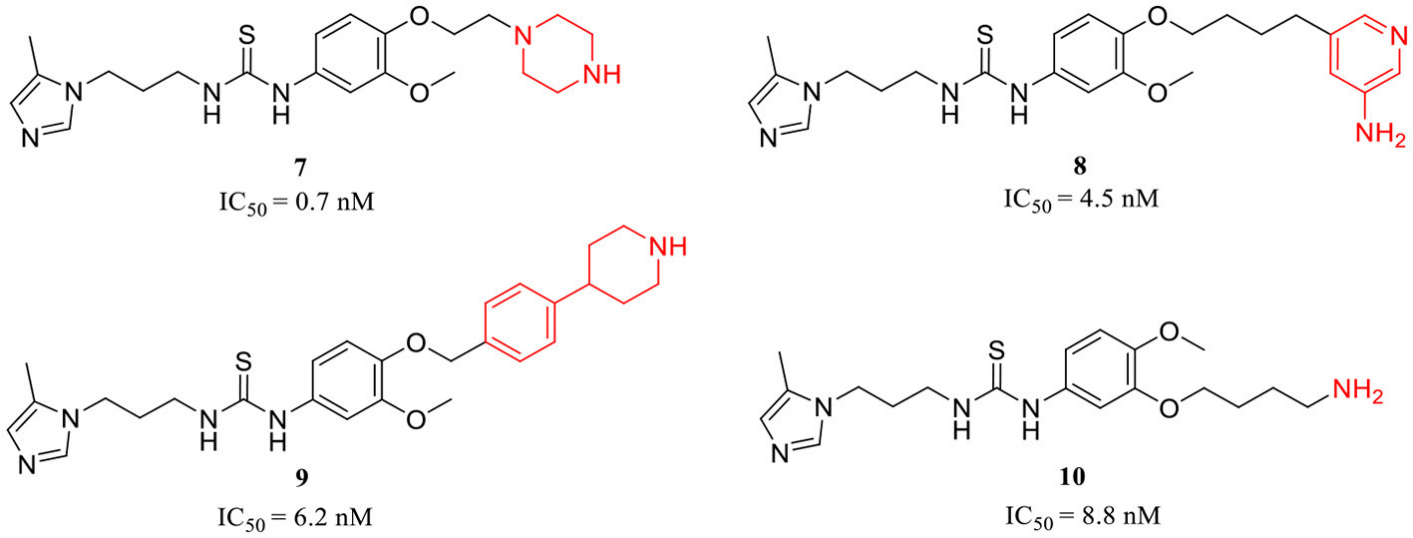	**7**, **8** (Hoang et al., 2017); **9** (Ngo et al., 2018a); **10** (Ngo et al., 2018b)
**(C) QCIs with restricted conformation**	
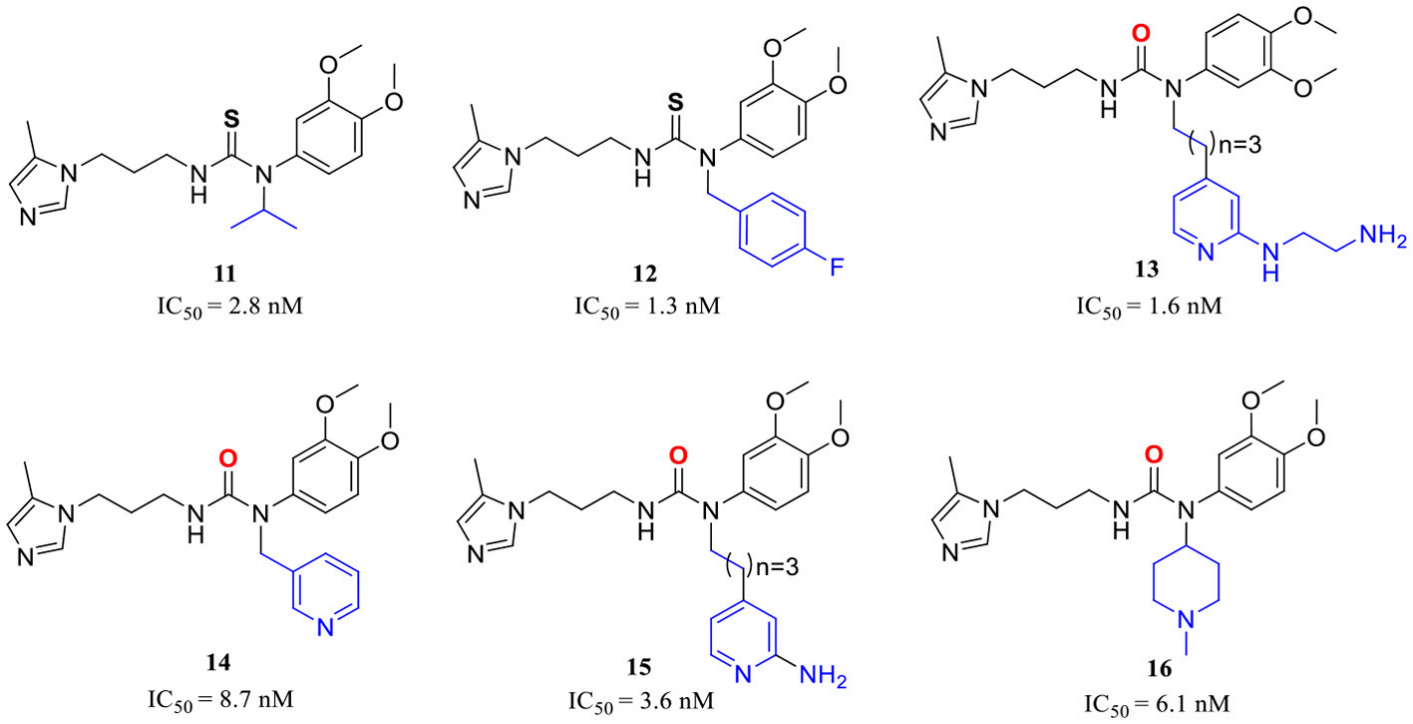	**11**–**16** (Hoang et al., 2019)
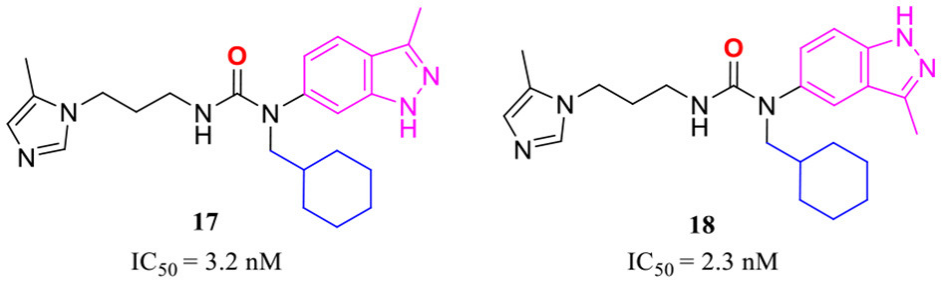	**17**, **18** (Van Manh et al., 2022)
**(D) QCIs with conformational blockers and extended motif D**	
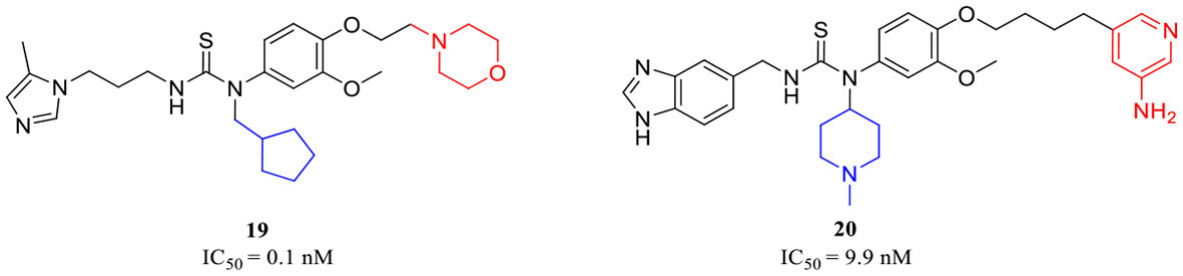	**19**, **20** (Van Manh et al., 2021)

**Figure 2:**
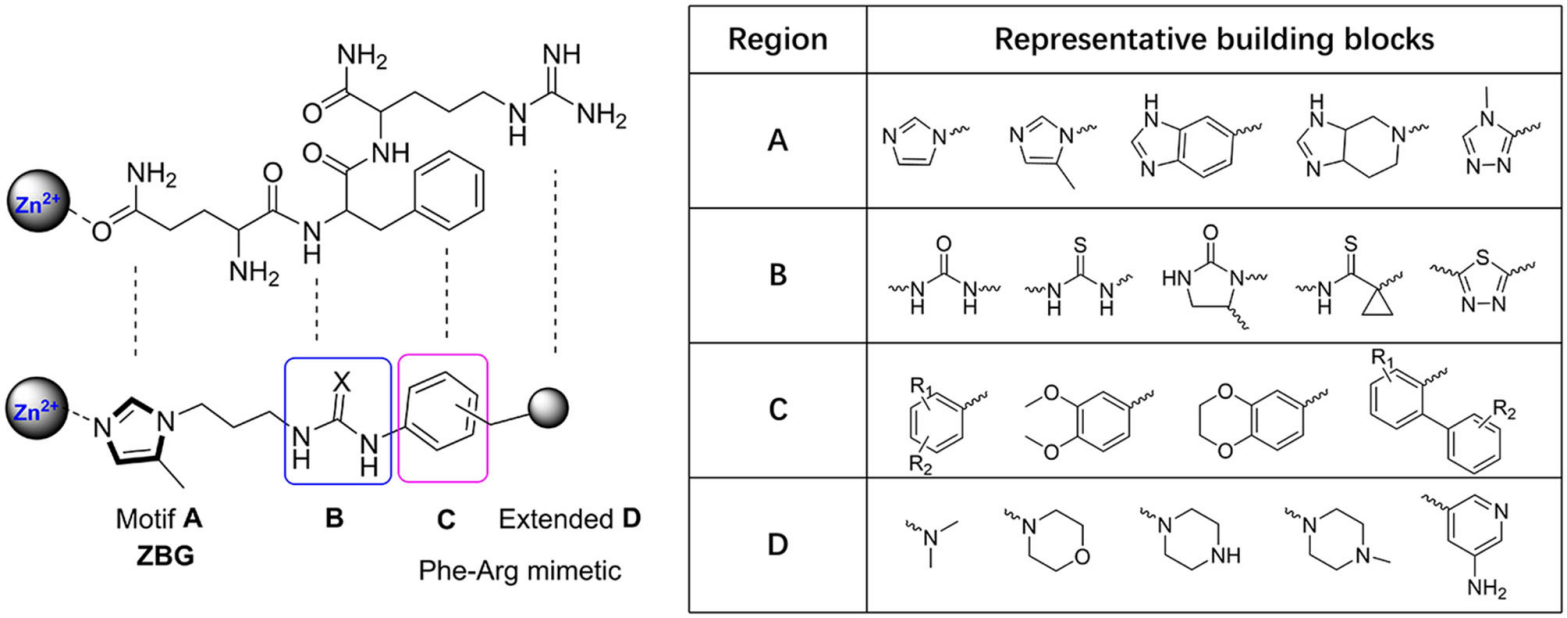
Essential pharmacophores of the classic QC inhibitor and its representative building blocks.

The classic framework of these inhibitors consists of three crucial motifs A, B, and C (Figure 2). The motif A contains a zinc-binding group (ZBG) or general metal-binding group (MBG), with imidazole, benzimidazole, and triazole as the most common structure entities. The imidazole-based ZBG, particularly the 5-methyl imidazole, is commonly the first choice (Kumar et al., 2013), leading to the fact that the imidazole-based inhibitors constitute the vast majority of the total QCIs. Recently, hydrazides were identified as the most potent ZBG compared with other classic Zn-binders (Kupski et al., 2020), which offer another option for designing novel inhibitors. The motif B contains at least a hydrogen bond donor (HBD) or a hydrogen bond acceptor (HBA); it is usually peptide amide analogs such as urea, thiourea, and their derivatives. Both urea and thiourea contributed not only more than one HBD and HBA but also flexible bonding (Tran et al., 2019). The motif C is normally an aromatic ring opposite or close to the ZBG, mimicking the Phe2 residue of Aβ_3 − 5_, which participates in the π-π interaction with the benzyl side chain of the essential Phe325 of QC. Among these classic inhibitors, PBD150 (Buchholz et al., 2006), **5** (PQ912) (Lues et al., 2015), and **6** (SEN177) (Jimenez-Sanchez et al., 2015) were the most outstanding representatives of imidazole, benzimidazole, and triazole-based QCIs, respectively (Table 1A). These inhibitors exhibit favorable pharmacodynamic profiles and are widely used as positive controls in numerous studies. However, only PQ912 is undergoing clinical trials until now.

The Phe2 and Arg3 residues of Aβ_3 − 5_ both are deeply involved in the interaction with QC. Nevertheless, the significant role of the guanidine side chain of Arg3 was underestimated earlier. To further mimic the feature of the guanidine group and to improve the QC inhibitory activity, Jeewoo Lee et al. added a nitrogen-containing heterocyclic group as an extended motif D based on the scaffold of **2** (Hoang et al., 2017). The newly developed inhibitors displayed 5- to 40-fold activity increase compared with **2** (IC_50_=29.2 nM in this assay). Though **7** (IC_50_=0.7 nM for hQC, Table 1B) was the most potent candidate even among previously reported inhibitors, it was found to be inactive in an acute ICR mice model to study the *in vivo* pE_3_-Aβ_40_ lowering efficacy. Whereas compound **8** (IC_50_ = 4.5 nM for hQC, Table 1B) exhibited a prominent efficacy of lowing pE_3_-Aβ_40_ by 54.7%, significantly reducing the brain pE_3_-Aβ_42_ level of APP/PS1 mice, and restoring the cognitive function of 5×FAD mice. Based on these encouraging results, the authors then systematically studied the SAR of **7** and **8** by modifying the Arg-mimetic motif, leading to the discovery of **9** (IC_50_ = 6.2 nM, Table 1B) (Ngo et al., 2018a) and **10** (IC_50_ = 8.8 nM, Table 1B) (Ngo et al., 2018b). Molecular modeling studies demonstrated that all these inhibitors formed extra strong salt bridge interactions with the carboxylate residue of Glu327, supporting the necessity of the extended motif D in high potent QC inhibitor design.

The X-ray structure showed that the PBD150 resided in the active site of hQC with a bent Z-E conformation (Huang et al., 2011; Hoang et al., 2019), while the 2-aminopyridine of extended D in **8** can freely rotate in the active site as revealed by molecular modeling (Hoang et al., 2017), suggesting the possibility of improving binding potency and inhibitory activity by a conformational restriction. Jeewoo Lee et al. creatively incorporated a conformational blocker into the urea or thiourea nitrogen of motif B to induce the formation of bent Z-E conformers (Hoang et al., 2019). The strategy was proven to be effective as well. 24 inhibitors with various rigid blocks showed a significant activity enhancement with *in vitro* IC_50_ below 10 nM compared with PBD150 (evaluated IC_50_ = 29.2 nM in this assay). The **11**, **12**, and **13** have a remarkably low IC_50_ value of 2.8 nM, 1.3 nM, and 1.6 nM, respectively, while the *in vivo* QC inhibition efficacy of these compounds was much weaker than that of **14** (IC_50_ = 8.7 nM), **15** (IC_50_=3.6 nM), and **16** (IC_50_ = 6.1 nM) (Table 1C), which suppressed the generation of pE_3_-Aβ_40_ by more than 20% in an acute mouse model compared with a negative control. Among the selected inhibitors, **16** exhibited the most promising *in vivo* efficacy and druggable profiles, such as liver microsomal stability and up to 50-fold inhibitory selectivity against gQC. The molecular docking further demonstrated that **16** displayed a Z-E conformation at the active site of QC, as anticipated. The N-substituted piperidinyl blocker of **16** not only restricted the conformation but also formed additional hydrophobic interactions with Tyr299, Val302, and Ile303, which may be highly correlated with the high inhibitory activity and QC selectivity. Remarkably, the SAR indicates that the effect of conformational restriction was more marked in the urea series than that of thiourea. In their recent study, the 3,4-dimethoxyphenyl group of the urea series scaffold was replaced by indazole bio-isosteres, which were regarded as more metabolically stable. The representative **17** and **18** (Table 1D), both containing an N-cyclohexylurea blocker, displayed remarkable inhibitory IC_50_ values of 3.2 and 2.3 nM, respectively (Van Manh et al., 2022).

Inspired by the encouraging results of both mimetic-Arg motif D and conformational restriction strategies on the classic QC scaffold. A combination of the two approaches was rationally performed, leading to the discovery of **19** (Table 1D), the most potent QC inhibitor reported even to date, with a sub-nanomolar IC_50_ value of 0.1 nM and up to 290-fold inhibitory enhancement compared with PQ912. While similar to the denouement of **12**, another weaker benzimidazole inhibitor **20** (IC_50_ = 9.9 nM, Table 1D) showed the most promising *in vivo* efficacy and selective profile with respect to its 21.5-fold sQC selectivity index toward gQC. Besides, **20** also has low toxicity and favorable pharmacokinetic properties, and it significantly improved the alternation behavior of mice in Y-maze tests as well.

Among these QCIs with high scaffold similarity, the common large polar groups such as urea and thiourea reduce the blood-brain barrier (BBB) permeability of the compounds, which may be the most likely reasons for the moderate or even inactive *in vivo* efficacy of the high potent inhibitors PBD150 (Brooks et al., 2015), **7**, **11**, **12**, **19**, etc. To ameliorate BBB permeability, Wu et al. tried to introduce a more hydrophobic biphenyl group in motif C to enhance molecular lipo-solubility as well as π-π stacking interaction and abandon the urea group in motif B (Li et al., 2017). The obtained **21** (Table 2A) exhibited potent inhibitory activity and significantly improved BBB permeability. Further assessments corroborated that **21** dramatically reduced the pE-Aβs level in cultured cells and *in vivo* and improved the behavior of B6C3-Tg AD mice. Interestingly, contrary to the commonly reported relationship, the SAR of DPCIs showed that 4-methyl substitution was better than that of 5-methyl substitution in imidazole. Although the activity of **21** was significantly decreased compared with the lead compound PBD150 due to the loss of the classic urea motif, the acquisition of SAR and the simple synthesis route of DPCIs still made it an ideal lead scaffold for further structural optimization.

**Table 2:** Other representative QCIs.

**(A) QCIs designed for improving BBB permeability**	
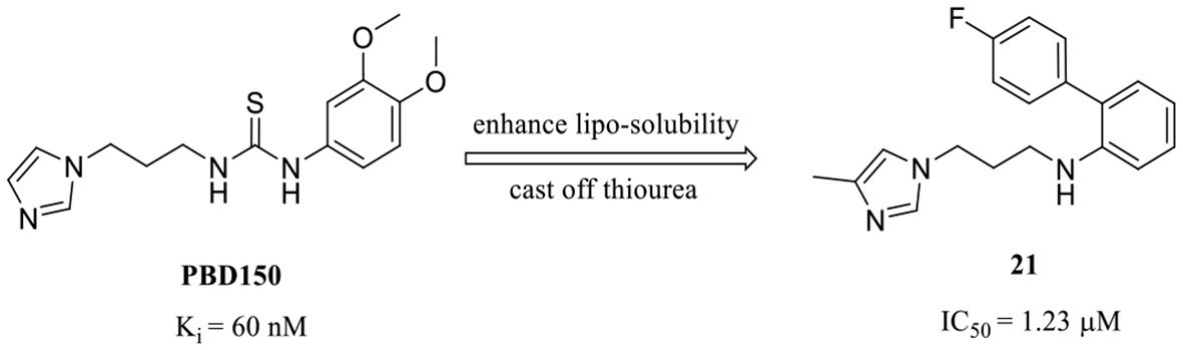	**21** (Li et al., 2017)
**(B) QCIs designed for non-hsQC with potent hsQC inhibitory activity**	
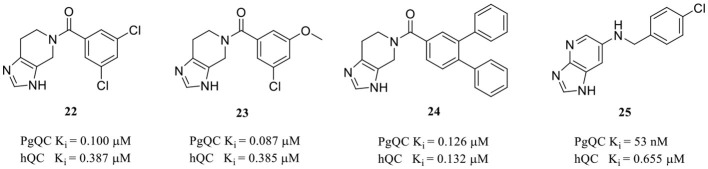	**22**–**24** (Ramsbeck et al., 2021); **25** (Taudte et al., 2021)
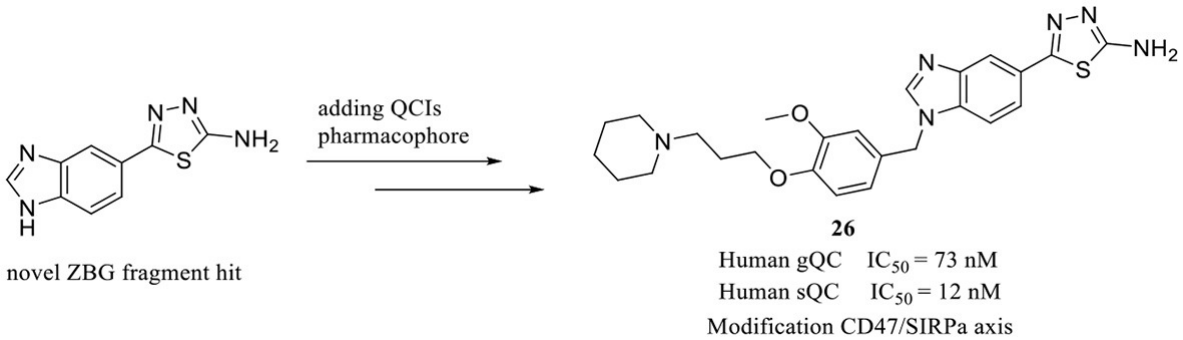	**26** (Park et al., 2022)

### 3.2. Representative QCIs designed for non-hsQC with potent hsQC inhibitory activity

Although the structural differences in both the overall conformations and active cores are essential and convenient for the design of type I/II QC-specific inhibitors, some bacterial QCs, such as *porphyromonas gingivalis* QC (PgQC), show similar structures and enzymatic features to hQC and were thus characterized as Mammalian-like type II QCs (Lamers et al., 2021). Therefore, inhibitors designed for bacterial QCs may also bind to and suppress hQC activity. For example, PgQC inhibitors **22**–**24** (Table 2B) designed for the treatment of periodontitis are almost equally potent in suppressing hQC activity *in vitro* (Lamers et al., 2021; Ramsbeck et al., 2021). Meanwhile, another inhibitor **25** with imidazo[4,5-b]pyridin scaffold exhibited a significantly improved selectivity (>12) over PgQC (Taudte et al., 2021).

Human gQC was recently recognized as an important modulator of the CD47-SIRPα pathway via promoting pGlu formation on the N-terminus of CD47 (Logtenberg et al., 2019). The gQC blockade contributes to reducing the “do not eat me” immune signals of CD47 on tumor cells. Therefore, developing gQC inhibitors is regarded as a novel and promising strategy for cancer immunotherapy. In a most recent study, a novel ZBG (1*H*-benzimidazol-5-yl)-1,3,4-thiadiazol-2-amine was hit by a fragment identified through library screening, and an aromatic ring and alkylamine were further added as additional QC pharmacophores. The most potent gQC inhibitor **26** (Table 2B) showed an outstanding IC_50_ of 73 nM, while unluckily it has 6-fold stronger activity against sQC with an IC_50_ of 12 nM (Park et al., 2022), which further indicates that more attentions should be paid to the selectivity of inhibitors when developing QCIs for the treatment of AD.

### 3.3. Virtual screening-based QCIs

In addition to the various rational design and experimental-based QCI discoveries, virtual screening offers another efficient tool to advance the understanding of activity profiles, and the development of new QCIs (Kumar et al., 2013; Lin et al., 2019). Those screening strategies include fragment-based screening (Szaszko et al., 2017), QSAR modeling (Al-Attraqchi and Venugopala, 2020; Kumar et al., 2021), and pharmacophore-assisted high-throughput virtual screening (Lin et al., 2019). Katharigatta N. Venugopala et al. developed linear and non-linear 2D QSAR models and a partial least squares-based 3D model to help predict the activity of not yet synthesized compounds. Combined with ADME filtering and 2D-similarity search, potential QCIs **27**–**29** (Table 3) were identified from the ZINC database (Al-Attraqchi and Venugopala, 2020). Similarly, Ashwani Kumar et al. identified the structural features that are both positively and negatively responsible for the QC inhibitory activity based on a dataset of 125 QCIs for QSAR analysis via Monte Carlo modeling studies. The QSAR further supports the importance of 5-methy substituted imidazole and alkyl-substituted benzene in activity enhancement, as previous SAR revealed, and novel compounds **30**–**32** (Table 3) were then computationally designed and showed improved *pKi* and QC binding affinities (Kumar et al., 2021). The hits of the two studies actually inherited typical features of classic QCIs with imidazole or methyl-imidazole as ZBG and an aromatic group located in the opposite position, while the QC inhibitory activities were not experimentally evaluated and validated *in vitro*.

**Table 3:** Representative screening-based QCIs.

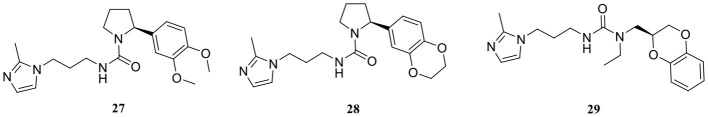	**27–29** (Al-Attraqchi and Venugopala, 2020)
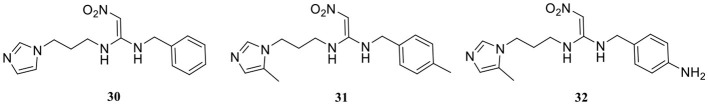	**30**–**32** (Kumar et al., 2021)
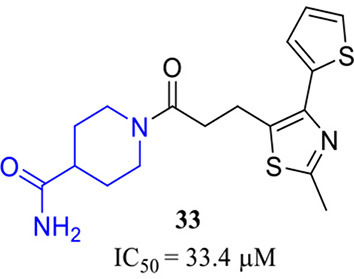	**33** (Dileep et al., 2021)
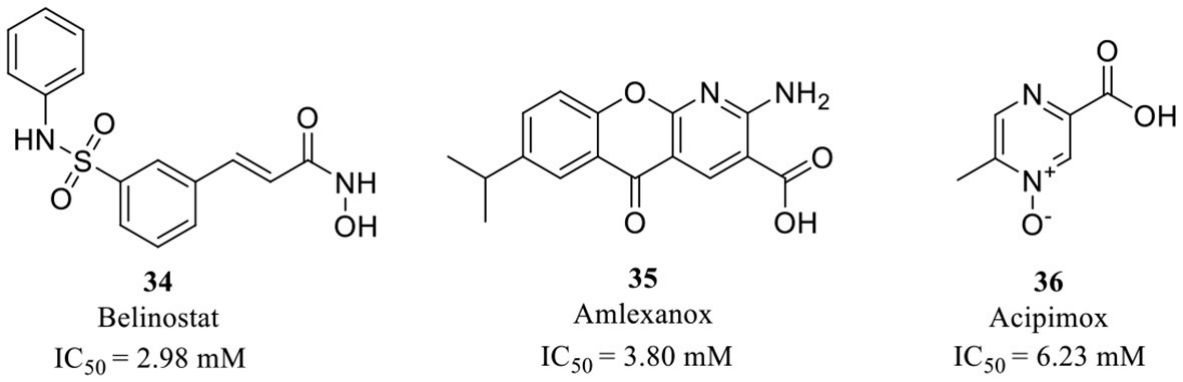	**34**–**36** (Xu et al., 2020)

Combining activity evaluation with virtual screening will provide more convincing evidence. Kam Y. J. Zhang et al. reported a QC inhibitor **33** (Table 3) with a novel MBG moiety, peperidine-4-carboxamide, through a pharmacophore-assisted high-throughput virtual screening (Dileep et al., 2021). **33** showed moderate activity against QC with IC_50_ = 33.4 ± 5.1 μM, and docking, MD simulation, and crystallographic studies suggested that **33** anchors to the active site via a coordinate bond with Zn^2+^ located deeply in the active site cleft of the QC, while it lacks stacking interactions with Tyr299, Phe325, and Trp329, which are assumed to be critical for QC activity.

Wu et al. performed a less efficient but simple and direct approach for new scaffold QCIs discovery by repurposing FDA-approved drugs (Xu et al., 2020). Such a repurposing strategy is more likely to succeed since the drugs have been fully evaluated in both pre-clinical and clinical trials. The QC inhibitory evaluation of 1,621 drugs was performed at a concentration of 10 μM *in vitro*, and the top five compounds were highlighted with reasonable activity. Although the inhibitory activity was relatively weak with IC_50_ values at the millimolar level, only two drugs contained the imidazole group; the other three drugs **34**–**36** (Belinostat, Amlexanox, and Acipimox; Table 3) have completely different structures from the classic QCIs model, which may still offer insights for the design and discovery of novel QCIs with new structural features.

### 3.4. Natural product-based QCIs

Imidazole- and benzimidazole-based ZBGs have so far been the first choice for the design of QC inhibitors. Nevertheless, these ZBG groups are less selective and are likely to interact with various metalloproteins *in vivo* and thus increase the risk of side effects (Park et al., 2022). Natural products are an important resource for the discovery of new activity scaffolds, which may offer opportunities to overcome the potential drawbacks of classic QCIs with new pharmacophores (Table 4). The oleuropein aglycone (OLE, **37**, Table 4), a natural phenol (secoiridoid) abundant in extra virgin olive oil, was found to be protective both in memory and behavioral performance of young and middle-aged TgCRND8 mice (Grossi et al., 2013). In an extended study of aged TgCRND8 mice showing increased pE_3_-Aβ_42_ deposits in the brain, OLE could also retard the growth of pE_3_-Aβ_42_ aggregates even in advanced and late stages of Aβ deposition (Luccarini et al., 2015). Several reviews summarized OLE as a QC inhibitor, while OLE certainly showed weak inhibitory activity at a concentration of 10 μM. Immunofluorescence staining and immunoblot analysis demonstrated that QC levels were significantly reduced in the brains of the OLE-fed Tg mice, suggesting that OLE is active against pE-Aβ generation by reducing QC expression rather than direct inhibition (Luccarini et al., 2015).

**Table 4:** Natural compounds with potential QC inhibitory activity.

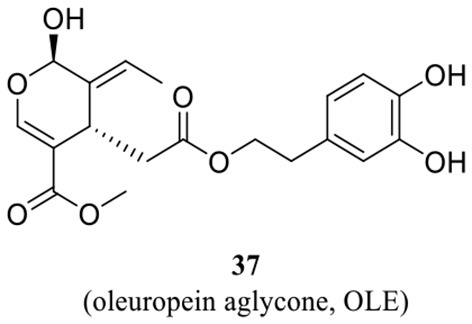	**37** (Luccarini et al., 2015) and CAS: 31773-95-2
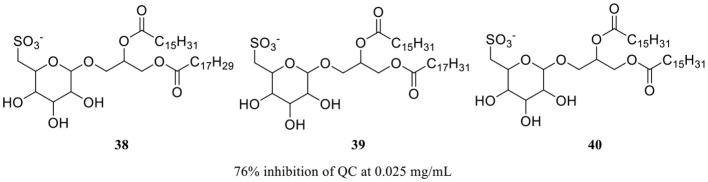	**38**–**40** (Hielscher-Michael et al., 2016)
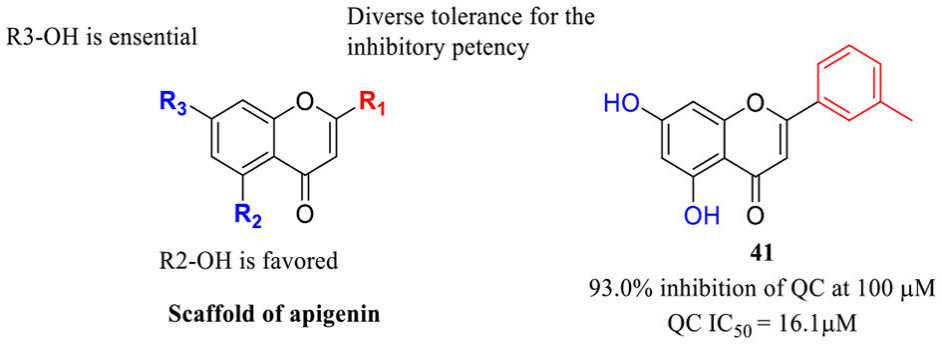	**41** (Li et al., 2016)

Some algal extracts were preliminary reported to have positive effects on QC inhibition, while the bioactive chemical entities were not isolated and clearly identified by traditional methods, Wessjohann et al. identified three sulfolipid QC inhibitors (**38**–**40**, Table 4) from microalgae using a new “Reverse Metabolomics” technique including an activity-correlation analysis. The sulfolipids showed a noteworthy QC inhibition of 76% at a low concentration of 0.025 mg/ml, and the authors proposed that sulfolipids provide similar pharmacophore characteristics to PBD150, in which the negative sulfonate group and the polyhydroxy elements probably act as a ZBG and the glucose as the core scaffold. Interestingly, SODG (structure not shown), a lipid product used as a standard reference in the assay, was first shown to exhibit quite similar QC inhibition activity compared with sulfolipids (Hielscher-Michael et al., 2016). Unlike traditional natural product screening approaches, Wu et al. explored apigenin-based QCI discovery via chemical modification. A total of 40 apigenin derivatives belonging to the phenol-4′(R1), C5-OH(R2), and C7-OH(R3) modified series were synthesized and evaluated. The compound **41** (Table 4) has remarkable inhibitory potency with an IC_50_ value of 16.1±2 μM, and the SAR study indicated that the C7-OH was required for binding with Zn^2+^ and that the C5-OH was favored, whereas phenol-4′ was tolerant for the inhibitory activity. The essential role of C7-OH was further supported by the binding interaction with conservative Zn^2+^ via molecular docking. Although the activity of apigenin derivates was relatively weak compared with nanomolar level classic QCIs, the non-imidazole ZBG, acquisition of SAR, and simple synthesis route made it a potential lead scaffold for further optimization (Li et al., 2016).

## 4. QC inhibitor undergoing clinical trials

Hundreds of QC inhibitors have been revealed in the literature, and reports of novel, high-potency QC inhibitors have dramatically increased in recent years with the structure-activity relationships becoming clear, while only PQ912 is currently undergoing clinical trials in human subjects for AD treatment. PQ912 has a scaffold slightly different from the common classic QCIs (Table 1A). It is a heterocyclic competitive inhibitor with benzimidazole as the ZBG at position 1 of the imidazolidine-2-one. PQ912 has strong human, rat, and mouse QC inhibitory activity with K_i_ values ranging between 20 and 65 nM (Hoffmann et al., 2017). Preclinical studies revealed that PQ912 has an attractive drug-like profile and robust pharmacological therapeutic effects, both *in vitro* and *in vivo* (Hoffmann et al., 2017). PQ912 was considered safe and well tolerated with dose-proportional pharmacokinetics up to doses of 200 mg in the first-in-man phase 1 study (Lues et al., 2015). In the subsequent randomized, double-blind, placebo-controlled phase IIa trial (NCT 02389413), the safety, tolerability, and efficacy of higher doses of PQ912 (800 mg twice daily for 12 weeks) were carefully evaluated in biomarkers confirmed early AD patients (*n* = 120). PQ912 showed an acceptable safety and tolerability profile in a treatment regime of lower doses and slower titration (Scheltens et al., 2018). The treatment of PQ912 resulted in an average QC target occupancy of >90% in cerebrospinal fluid (CSF), an improvement of working memory, and a reduction of synaptotoxicity and neurogranin levels, as well as improvements in some other experimental endpoints (Scheltens et al., 2018). However, no PQ912 treatment effects were found on the composite scores of episodic memory, executive function, attention, or overall cognition (Scheltens et al., 2018). The results of the phase IIa study might indicate early beneficial effects of PQ912 on cognition by preventing the synaptotoxicity of pE-Aβ in the central nervous system and thus rescuing impaired synaptic functions. While episodic memory is the most impaired area of function in AD pathology, a relatively short intervention (12-week time period) by PQ912 was actually not expected to have an obvious clinical influence on episodic and long-term memory. To further test the efficacy (particularly on cognition and brain activity) of PQ912 as a disease modifier, a phase IIb program in participants with MCI and mild AD-VIVIAD was recently launched, and results are expected early in 2023 (Vijverberg et al., 2021).

## 5. New trends toward QCIs

Theoretically, QC inhibition can significantly decrease the formation of pE-Aβ while having little influence on the clearance of full-length Aβ and the existing pE-Aβ, which may still induce Aβ cascades and lead to the deposition of senile plaques. Hence, QC inhibition-based combination therapy and multi-target-directed ligands (MTDL) have drawn considerable attentions in recent years. The combination effects of PQ912 and a pE-Aβ specific antibody m6 on the formation and clearance of pE-Aβ in an AD mouse model were evaluated. The study showed that combination treatments resulted in significant reductions of total Aβ by 45–65% in the brain of AD mouse overexpressing both human amyloid precursor protein containing the Swedish and London mutations and human QC (hAPPsl × hQC), while single treatments at subtherapeutic levels only showed a moderate (16–41%) but statistically insignificant reduction in Aβ level. The additive effects of the combination of PQ912 and m6 on brain Aβ pathology were evaluated using a bliss independence model, and a combination index of ≈1 was determined (Hoffmann et al., 2021). The combination strategy may achieve a better therapeutic effect than a single treatment, even at a reduced dose for the individual drug.

Instead of drug combination treatment, Wu et al. in their recent study developed a new class of maleimide-DPCI hybrid QC/GSK-3β dual inhibitors by rationally combining the essential pharmacophores of QC and GSK-3β inhibitors. GSK-3β, namely, glycogen synthase kinase-3, is regarded as a critical pivotal kinase and a high-potential anti-AD target that links both Aβ and tau pathologies of AD. The most potent compounds **42**–**44** (Table 5) exhibited slightly enhanced QC inhibitory activity and similar GSK-3β inhibitory activity compared with individual control compounds DPCI-2 and SB-415286, respectively. The selected dual-target inhibitor **42** can dramatically reduce pE-Aβ accumulation and Tau hyperphosphorylation in the brains of 3 × Tg-AD mice. In addition, **42** also effectively attenuates cognitive deficits and decreases anxiety-like behavior in 3 × Tg mouse (Xie et al., 2023).

**Table 5:** Dual-target QCIs.

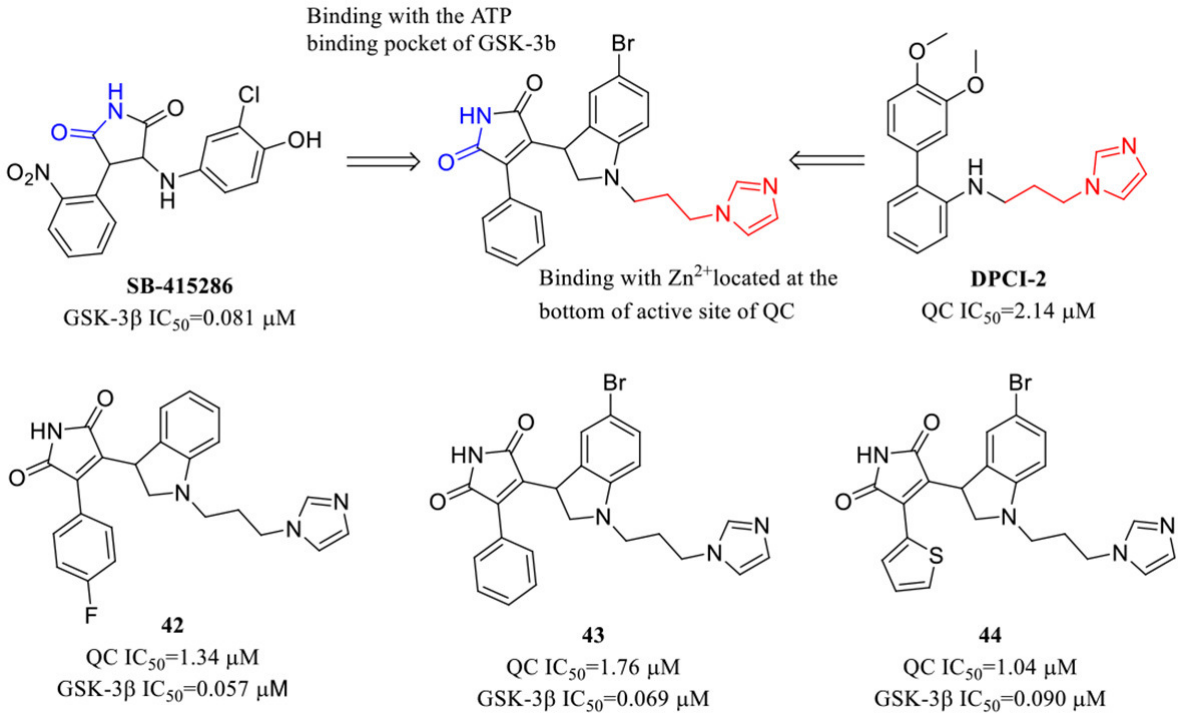	**42**–**44** (Xie et al., 2023)

## 6. Perspective and conclusion

pE_3_-Aβ represents a highly desirable and abundant target due to its distinctive aggregation properties and neurotoxicity. As QC plays a key role in the conversion and formation of pE_3_-Aβ, QC inhibition is emerging as an alternative promising strategy apart from expensive active immune clearance to decrease the pathological toxicity of pE_3_-Aβ for the treatment of AD. Over the past two decades, a number of different scaffold QCIs have been designed and discovered, and the efficacy of several high potent inhibitors has been evaluated both *in vitro* and *in vivo* using different AD mice models. Moreover, PQ912 is regarded as the proof-of-concept validation of the QC target. However, there are still issues to be considered before QC inhibitors can effectively translate from bench to bedside.

First, the specificity of QC inhibitors, which includes the selective inhibition of hQC among various metalloproteins *in vivo* and selectivity toward the QC-Aβ pathway rather than other QC normal PTMs processes. Because QC is abundant in mammalian neuroendocrine tissues and is responsible for the maturation of numerous hormones and cytokines, non-selective QC inhibition may lead to wide and unpredictable side effects. In the phase IIa trial of PQ912, one-third (20/60) of subjects discontinued PQ912 treatment due to adverse events related to gastrointestinal disorders, skin disorders, etc. A broader battery of CSF biomarkers, including growth-associated protein 43 and pE-CCL2, have thus been set as exploratory endpoints to better monitor the potential adverse effects of the treatment in AD patients. Second, the pathological reversing effects of QC enzyme inhibitors remain questionable. Given that pE-Aβ acts as seeds to induce the formation of stable toxic heterogeneity polymers, QC inhibition can suppress but not completely prevent the pE-Aβ formation. The existing pE-Aβ may still trigger Aβ cascades. Fortunately, the QC-based combination strategy and MTDL strategy are drawing attention lately, which might be an ideal solution to enhance the additive effects. Last but not least, the pE-Aβ cascade is essentially an optimization of the original Aβ cascade hypothesis, in which the pE-Aβ replaces full-length Aβ serving as the core initiator in the progression of AD. In the context of endless clinical failures of Aβ-directed or related interventions and the controversy regarding the exact role of Aβ cascade in the pathogenesis of AD, how far will QC inhibition go remains blurred in clinical trials. Nevertheless, the encouraging effect of the pE-Aβ-targeting donanemab in clinical trials envisions the promising role of the pE-Aβ-related key protein QC as an alternative potential target for the novel disease-modifying treatment of AD.

## Author contributions

All authors listed have made a substantial, direct, and intellectual contribution to the work and approved it for publication.
